# Construction of industrial *Saccharomyces cerevisiae* strains for the efficient consolidated bioprocessing of raw starch

**DOI:** 10.1186/s13068-019-1541-5

**Published:** 2019-08-20

**Authors:** Rosemary A. Cripwell, Shaunita H. Rose, Lorenzo Favaro, Willem H. van Zyl

**Affiliations:** 1 Department of Microbiology, Stellenbosch University, Private Bag X1, Matieland, 7602 South Africa; 2 Department of Agronomy Food Natural resources Animals and Environment (DAFNAE), Università di Padova, Agripolis, Viale dell’Università 16, 35020 Legnaro, Padova Italy

**Keywords:** Consolidated bioprocessing, Biofuels, Industrial yeast, Amylases, Raw corn starch, *Talaromyces emersonii*

## Abstract

**Background:**

Consolidated bioprocessing (CBP) combines enzyme production, saccharification and fermentation into a one-step process. This strategy represents a promising alternative for economic ethanol production from starchy biomass with the use of amylolytic industrial yeast strains.

**Results:**

Recombinant *Saccharomyces cerevisiae* Y294 laboratory strains simultaneously expressing an α-amylase and glucoamylase gene were screened to identify the best enzyme combination for raw starch hydrolysis. The codon optimised *Talaromyces emersonii* glucoamylase encoding gene (*temG_Opt*) and the native *T. emersonii* α-amylase encoding gene (*temA*) were selected for expression in two industrial *S. cerevisiae* yeast strains, namely Ethanol Red™ (hereafter referred to as the ER) and M2n. Two δ-integration gene cassettes were constructed to allow for the simultaneous multiple integrations of the *temG_Opt* and *temA* genes into the yeasts’ genomes. During the fermentation of 200 g l^−1^ raw corn starch, the amylolytic industrial strains were able to ferment raw corn starch to ethanol in a single step with high ethanol yields. After 192 h at 30 °C, the *S. cerevisiae* ER T12 and M2n T1 strains (containing integrated *temA* and *temG_Opt* gene cassettes) produced 89.35 and 98.13 g l^−1^ ethanol, respectively, corresponding to estimated carbon conversions of 87 and 94%, respectively. The addition of a commercial granular starch enzyme cocktail in combination with the amylolytic yeast allowed for a 90% reduction in exogenous enzyme dosage, compared to the conventional simultaneous saccharification and fermentation (SSF) control experiment with the parental industrial host strains.

**Conclusions:**

A novel amylolytic enzyme combination has been produced by two industrial *S. cerevisiae* strains. These recombinant strains represent potential drop-in CBP yeast substitutes for the existing conventional and raw starch fermentation processes.

## Background

Starch is a readily available renewable material found in most regions of the world [1]. There are numerous types of starchy biomass that represent attractive substrates for bioethanol production, namely corn (maize), wheat, oats, rice, potato and cassava [2]. For decades, amylolytic enzymes from various microbial sources have been used in starch based processes, which has led to amylases being among the most important enzymes used for industrial applications [3]. However, only a limited number of fungal and bacterial strains meet the criteria for commercial amylase production. Therefore, new microorganisms are continuously screened for amylase activity, especially for applications in the biofuel industry.

The conventional process for the conversion of starch to ethanol requires a heat-intensive liquefaction step together with thermostable α-amylases to gelatinise the starch, followed by saccharification with a glucoamylase. The high temperatures required for the initial processes usually account for approximately 30–40% of the total energy required for ethanol production [4]. An alternative to this is a cold hydrolysis process at temperatures below the onset of starch gelatinisation (65 °C for corn) [5]. Benefits of this process include reduced energy requirements and higher nutritional content for the distiller’s dried grains with solubles (DDGS) [6]. DDGS are produced in large quantities during bioethanol production and represent a valuable ingredient for livestock feed [7].

Consolidated bioprocessing (CBP) using a single organism combines enzyme production, substrate hydrolysis and glucose fermentation into a one-step process for bioethanol production at low temperatures [8]. This technology has developed rapidly over the last decade and is a promising approach for the economic production of biofuel from lignocellulosic and starchy feedstocks [9]. However, CBP has not yet been implemented on a commercial scale with the main challenge being the availability of an ideal host microorganism that can express suitable enzymes and have a high fermentation capacity [10]. CBP would simplify operational processes (e.g. number of control steps and reaction vessels) and, therefore, reduce maintenance and production costs. The comprehensive review on consolidated bioprocessing systems [9] highlighted different CBP strategies, diversity in the substrate types, as well as the organisms involved in fermenting the sugars.

Currently, no industrial process uses a recombinant amylolytic yeast strain for starch CBP that produces both an α-amylase and glucoamylase, but the commercial production of granular starch hydrolysing enzyme (GSHE) cocktails has allowed for the development of simultaneous saccharification and fermentation (SSF) processes (at lower temperatures) for ethanol production from starchy substrates [4, 6]. An existing market is available for a drop-in CBP yeast that is able to simultaneously express raw starch α-amylase and glucoamylase encoding genes for complete starch hydrolysis. One of the main challenges remains the simultaneous production of these enzymes exhibiting high substrate affinities and specific activity [11].

It is estimated that the use of raw starch hydrolysing enzymes for ethanol production reduces energy costs by 10–20% [5]. Currently, the commercially available GSHE cocktails from DuPont Industrial Biosciences (DuPont-Danisco, Itasca, USA) hydrolyse raw starch at low temperatures (48 °C is recommended for SSF), while POET (Sioux Falls, South Dakota, USA) use a patented blend of Novozymes enzymes (POET BPX technology) in an SSF process [12]. Glucoamylase producing *Saccharomyces cerevisiae* strains such as TransFerm^®^ (Lallemand, Montreal, Canada) and Innova^®^Drive (Novozymes, Copenhagen, Denmark) are commercially available (http://www.ethanoltech.com/transferm and https://www.novozymes.com/en/advance-your-business/bioenergy/innovadrive, respectively). However, these recombinant yeast strains lack an α–amylase enzyme required for starch liquefaction [10, 13] and are therefore only semi-CBP yeast. Recently, amylase corn (corn containing endogenous α-amylase) in combination with a “superior” glucoamylase producing yeast strain has been used to improve ethanol yields [14], but this process still requires high temperatures (85 °C) for starch gelatinisation.

In this study, novel amylase gene combinations were cloned and expressed in the *S. cerevisiae* Y294 laboratory strain and the amylolytic transformants were screened for their raw starch fermenting ability. Subsequently, the enzyme combination that best hydrolysed raw corn starch was expressed in two industrial *S. cerevisiae* strains, namely Ethanol Red™ which is one of the most widely used yeast strains for first-generation bioethanol production [15] and M2n, which is a South African distillery yeast [16]. Gene integration and the acetamide selection method were used for the engineering of the industrial yeast strains. The use of the acetamidase encoding gene (*amdS*) as a dominant marker enabled the selection of recombinant prototrophic strains on acetamide [17], which replaced the conventional selection method that requires antibiotics. The industrial amylolytic strains were evaluated at high solids loadings under two different fermentation temperatures and were able to convert raw corn starch into ethanol, with a yield close to the theoretical maximum.

## Results and discussion

### Cloning and recombinant amylase expression in *S. cerevisiae* Y294

**Fig. 1:**
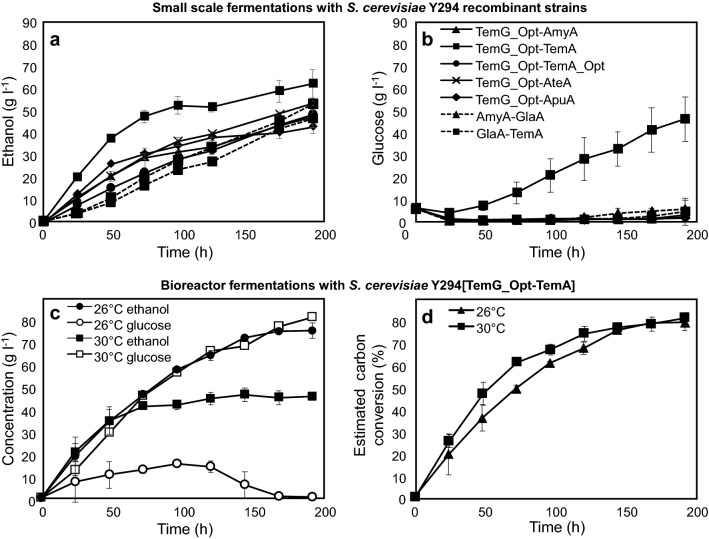
The amylolytic *S. cerevisiae* Y294 strains were evaluated at 30 °C under oxygen-limited conditions in 100 ml fermentation bottles containing 2 × SC^–URA^ broth supplemented with 5 g l^−1^ glucose and 200 g l^−1^ raw corn starch as carbohydrate sources. The **a** ethanol and **b** glucose production was monitored overtime. The *S. cerevisiae* Y294[TemG_Opt-TemA] strain was cultivated in a 2-l bioreactor, **c** ethanol and residual glucose concentrations at 26 and 30 °C, and **d** the percentage estimated carbon conversion at 26 and 30 °C. Values represent the mean of three repeats and error bars represent the standard deviation

Starch-rich biomass is currently the main substrate for bioethanol production in the United States [18] and it can be efficiently hydrolysed using α-amylase and glucoamylase enzymes [19]. Following the identification of novel amylase candidates with superior hydrolytic activity [20] several plasmids were designed to simultaneously express two amylase genes, namely an α-amylase and glucoamylase gene combination, under the transcriptional control of the *ENO1* promoter and terminator sequences. The episomal plasmids were introduced into the *S. cerevisiae* Y294 laboratory strain to obtain amylolytic yeasts suitable for the one-step conversion of raw corn starch flour (henceforth referred to as raw corn starch) to ethanol. The recombinant strains (listed in Table 1) were evaluated for their ability to hydrolyse raw corn starch at a high substrate loading (200 g l^−1^ corn starch) and ferment the resulting glucose to ethanol. Six different recombinant amylolytic strains, expressing novel gene combinations, were compared to the previously constructed *S. cerevisiae* Y294[AmyA-GlaA] strain [19], which presented as benchmark yeast for this screening process (Fig. 1a, b).

**Table 1:** Strains and plasmids used in this study

Strains and plasmids	Genotype	References/source
*E. coli DH5α*	*supE44 ΔlacU169 (ϕ80lacZ*ΔM15*) hdR17 recA1 endA1 gyrA96 thi*-*1 relA1*	[43]
*S. cerevisiae* strains		
Y294	*α leu2*-3,112 *ura3*-52 *his3 trp1*-*289*	ATCC 201160
Y294[AmyA-GlaA]	*URA3 ENO1*_P_-*glaA*-*ENO1*_T_; *ENO1*_P_-*amyA*-*ENO1*_T_	[19]
Y294[GlaA-TemA]	*URA3 ENO1*_P_-*glaA*-*ENO1*_T_; *ENO1*_P_-*temA*-*ENO1*_T_	This study
Y294[TemG_Opt-AmyA]	*URA3 ENO1*_P_-*temG_Opt*-*ENO1*_T_; *ENO1*_P_-*amyA*-*ENO1*_T_	This study
Y294[TemG_Opt-AteA]	*URA3 ENO1*_P_-*temG_Opt*-*ENO1*_T_; *ENO1*_P_-*ateA*-*ENO1*_T_	This study
Y294[TemG_Opt-ApuA]	*URA3 ENO1*_P_-*temG_Opt*-*ENO1*_T_; *ENO1*_P_-*apuA*-*ENO1*_T_	This study
Y294[TemG_Opt-TemA]	*URA3 ENO1*_P_-*temG_Opt*-*ENO1*_T_; *ENO1*_P_-*temA*-*ENO1*_T_	This study
Y294[TemG_Opt-TemA_Opt]	*URA3 ENO1*_P_-*temG_Opt*-*ENO1*_T_; *ENO1*_P_-*temA_Opt*-*ENO1*_T_	This study
Y294[amdSYM]	*URA3 TEF*_P_-*amdS*-*TEF*_T_	This study
ER^a^	*MATa/α* prototroph	Fermentis, Lesaffre, France
M2n	*MATa/α* prototroph	[16]
ER T1^b^	δ-integration of *ENO1*_P_-*temG_Opt*-*ENO1*_T_; *ENO1*_P_-*temA*-*ENO1*_T_	This study
ER T12^b^	δ-integration of *ENO1*_P_-*temG_Opt*-*ENO1*_T_; *ENO1*_P_-*temA*-*ENO1*_T_	This study
M2n T1^b^	δ-integration of *ENO1*_P_-*temG_Opt*-*ENO1*_T_; *ENO1*_P_-*temA*-*ENO1*_T_	This study
M2n T2^b^	δ-integration of *ENO1*_P_-*temG_Opt*-*ENO1*_T_; *ENO1*_P_-*temA*-*ENO1*_T_	This study
Plasmids		
yBBH1	*bla URA3 ENO1*_P_-*ENO1*_T_	[49]
yBBH1-AmyA	*bla URA3 ENO1*_P_-*amyA*-*ENO1*_T_	[19]
yBBH1-GlaA	*bla URA3 ENO1*_P_-*glaA*-*ENO1*_T_	[19]
yBBH1-AteA	*bla URA3 ENO1*_P_-*ateA*-*ENO1*_T_	[20]
yBBH1-ApuA	*bla URA3 ENO1*_P_-*apuA*-*ENO1*_T_	[20]
yBBH1-TemA^c^	*bla URA3 ENO1*_P_-*temA*-*ENO1*_T_	[20]
yBBH1-TemA_Opt^c^	*bla URA3 ENO1*_P_-*temA_Opt*-*ENO1*_T_	[20]
yBBH1-TemG_Opt^d^	*bla URA3 ENO1*_P_-*temG_Opt*-*ENO1*_T_	[20]
yBBH1-TemG_Opt-TemA	*bla URA3 ENO1*_P_-*temG_Opt*-*ENO1*_T_; *ENO1*_P_-*temA*-*ENO1*_T_	This study
pUG-amdSYM^e^	*bla TEF1*_P_-*amdS*-*TEF1*_T_	[17]
yBBH1-amdSYM	*bla URA3 TEF1*_P_-*amdS*-*TEF1*_T_	This study

^a^Ethanol Red™ Version 1, referred to as ER

^b^Amylolytic transformants contain integrated copies of *ENO1*_P_-*temA*-*ENO1*_T_ and *ENO1*_P_–*temG_Opt*-*ENO1*_T_ gene cassettes, the number indicates the transformant number during the screening process

^c^Accession no. XM_013469492 for the native *Talaromyces emersonii* α-amylase (*temA*—1866 bp/622 amino acids)

^d^Accession no. AJ304803 for the native *T. emersonii* glucoamylase (*temG*—1854 bp/618 amino acids)

^e^Assession no. P30669 for pUG-amdSYM plasmid

After 120 h, the Y294[TemG_Opt-TemA] strain had produced 51.71 g l^−1^ ethanol, which represented a 1.6-fold improvement on the Y294[AmyA–GlaA] benchmark strain, producing 33.14 g l^−1^ ethanol (*p* = 0.0013). Ethanol concentrations of 38.57 and 39.40 g l^−1^ produced by the Y294[TemG_Opt–ApuA] and Y294[TemG_Opt–AteA] strains (expressing the *Aureobasidium pullulans apuA* and *Aspergillus terreus ateA* α-amylases), respectively, were also higher than the benchmark strain at 120 h. At 192 h, the superior Y294[TemG_Opt-TemA] strain expressing the native *temA* α-amylase and codon-optimised *temG_Opt* glucoamylase (both genes originating from *T. emersonii*) accumulated the highest ethanol concentration (62.20 g l^−1^), which was 60% of the theoretical ethanol yield (Fig. 1a, Table 2). However, the non-robust nature of the Y294 strain resulted in an incomplete fermentation and, as a consequence, the Y294[TemG_Opt–TemA] strain accumulated 46.30 g l^−1^ residual glucose after 192 h of fermentation (Fig. 1b and Table 2). Nevertheless, the unfermented glucose highlighted that the TemG_Opt-TemA enzyme combination efficiently hydrolysed raw corn starch and noticeably separated this strain’s hydrolysing ability from the other recombinant strains, the latter resulted in insignificant residual glucose concentrations (< 6 g l^−1^) at the end of the fermentation (Fig. 1b).

**Table 2:** Product formation by the *S. cerevisiae* Y294 strains after 192 h of fermentation at 30 °C in 2 × SC^−URA^ broth containing glucose (5 g l^−1^) and raw corn starch (200 g l^−1^), as carbon sources, as well as starch hydrolysing activities (U ml^−1^) when strains were grown in 2 × SC^−URA^ broth for 72 h

*S. cerevisiae* Y294 strains	[TemG_Opt-AmyA]	[TemG_Opt-TemA]	[TemG_Opt-TemA_Opt]	[TemG_Opt-AteA]	[TemG_Opt-ApuA]	[GlaA-TemA]	[AmyA-GlaA]
Substrate (g l^−1^)							
Raw starch	200	200	200	200	200	200	200
Glucose equivalent	208.5	208.5	208.5	208.5	208.5	208.5	208.5
Products (g l^−1^)							
Glucose	2.72	46.30	1.67	1.94	1.21	4.12	5.30
Glycerol	4.76	6.64	2.40	3.43	2.45	2.26	2.46
Maltose	1.09	1.03	1.07	1.14	0.95	1.17	1.02
Acetic acid	1.91	1.66	0.60	0.85	0.61	0.56	0.61
Ethanol	47.40	62.20	48.71	53.46	43.12	46.56	52.78
CO_2_^a^	45.33	59.50	46.59	51.13	41.25	44.53	50.48
Total	103.21	177.33	101.04	111.95	89.60	99.20	112.65
Estimated carbon conversion (%)	50	85	49	54	43	48	54
Ethanol yield^b^ (% of theoretical yield)	46	60	47	51	41	45	51
Ethanol productivity^c^	0.25	0.32	0.25	0.28	0.23	0.24	0.28
2% raw starch assays (U ml^−1^)							
Total amylase activity^d^	0.21	0.47	0.16	0.21	0.30	0.13	0.09
Released glucose^e^	0.11	0.25	0.06	0.10	0.08	0.03	0.03
Released glucose equivalents^f^	0.16	0.43	0.19	0.35	0.32	0.05	0.05
0.2% soluble starch assays (U ml^−1^)							
Total amylase activity^d^	3.30	3.69	2.27	1.90	3.44	2.15	1.84
Released glucose^e^	0.69	1.00	0.16	0.32	0.13	0.01	0.02

The assays were performed at 30 °C in citrate buffer at pH 5 with raw and soluble corn starch

^a^CO_2_ concentrations were deduced from the ethanol produced

^b^Ethanol yield (% of the theoretical yield) was calculated as the amount of ethanol produced per gram of available glucose (at a specific time point)

^c^Ethanol productivity was calculated based on ethanol concentrations produced per h (g l^−1^ h^−1^)

^d^Reducing sugar assay detects all reducing sugars (monosaccharides and oligosaccharides)

^e^Glucose kit assay detects only glucose

^f^HPLC detection of glucose and maltose after raw starch assay, released glucose equivalents were converted to activity

There are a number of factors commonly associated with an incomplete fermentation, including the yeast strain’s background and nitrogen availability [21]. Fermentation temperature control and rising ethanol concentrations may lead to fermentation arrest [22] and, therefore, fermentation temperature is considered as one of the main parameters with regards to ethanol production by SSF and CBP strategies. Moreover, the effect of high temperature is intensified by ethanol concentrations, which can significantly affect the yeast’s fermenting capability [21]. When the cultivation temperature increases above the optimum growth temperature, the specific glucose uptake by *S. cerevisiae* is affected by changes to the physiology of the yeast cells and changes in the cell’s membrane [22, 23] and this might explain the high glucose residual concentration observed in the Y294[TemG_Opt–TemA_Opt] fermentation broth (Fig. 1b).

The estimated carbon conversion of 85% displayed by the *S. cerevisiae* Y294[TemG_Opt–TemA] was 31% higher than the Y294[TemG_Opt-AteA] strain, as well as the Y294[AmyA-GlaA] benchmark yeast (Table 2). The Y294[TemG_Opt–TemA] strain also showed an overall improvement in starch conversion to ethanol, compared to the previously constructed Y294[AteA-GlaA] strain [24], which produced 45.80 g l^−1^ ethanol after 144 h with an estimated carbon conversion of 45%.

The large difference in hydrolytic ability between recombinant strains could suggest that the specific TemG_Opt–TemA gene combination had enhanced synergistic activity for raw starch degradation, since even the Y294[TemG_Opt–TemA_Opt] strain, expressing the codon optimised *T. emersonii* α-amylase displayed significantly lower estimated carbon conversion and decreased starch hydrolysing activities (Table 2). The best performing Y294[TemG_Opt–TemA] strain displayed a total amylase activity of 0.47 U ml^−1^ on raw starch at 30 °C, while the next highest activity was displayed by the Y294[TemG_Opt–ApuA] strain (0.30 U ml^−1^). The data for released glucose equivalents from raw starch supported the results for total amylase activity, as well as the high residual glucose concentrations (46.30 g l^−1^) that were displayed by the Y294[TemG_Opt–TemA] strain under raw starch fermentation conditions (Table 2).

### *S. cerevisiae* Y294[TemG_Opt-TemA] bioreactor fermentations

Although *S. cerevisiae* is known for its ethanol tolerance, most strains still lack the ability to continue fermenting glucose at temperatures that are higher than their normal cultivation temperature (~ 30–34 °C for industrial strains, but lower for laboratory *S. cerevisiae* strains). The Y294[TemG_Opt-TemA] strain’s fermenting capability was thus compared using a 2-l benchtop bioreactor (1-l working volume) at both 26 and 30 °C, respectively (Fig. 1c, d). The optimum growth temperature for the Y294 strain is lower than 30 °C, therefore, a fermentation incubation temperature of 26 °C was included, as it more closely represents the ideal temperature for this laboratory *S. cerevisiae* strain’s fermenting ability (reported as 25 °C for the ATCC^®^ 201160™; https://www.atcc.org/~/ps/201160.ashx). After 192 h at 26 °C, a 1.8-fold improvement in the ethanol concentration (compared to at 30 °C) was detected and there was no residual glucose in the fermentation broth (Fig. 1c). From 144 h, the resulting estimated carbon conversion was similar for both fermentation temperatures (~ 80%) (Fig. 1d), demonstrating that the lower temperature did not negatively affect the starch hydrolysis and prevented the accumulation of unfermented glucose.

After 192 h at 30 °C, the estimated carbon conversion displayed by the *S. cerevisiae* Y294[TemG_Opt-TemA] strain was similar for the 100 ml and 1-l bioreactor fermentations, 85 and 81%, respectively (Table 2 and Fig. 1d). However, at 30 °C, ethanol levels obtained by *S. cerevisiae* Y294[TemG_Opt-TemA] were found to be lower than those detected at a smaller scale (Fig. 1a). This finding could be due to an increase in stress exposure linked to limited transportation and elimination of CO_2_, toxic metabolites and additional heat generated by agitation [25]. The effect of temperature on fermentation products has been previously observed by a number of different research groups [23, 26] and it is suggested that in this study a lower incubation temperature lessened the physiological stress on the cells and enabled the *S. cerevisiae* Y294[TemG_Opt-TemA] laboratory strain to ferment all the available glucose to ethanol. An additional factor that may have intensified the effect of fermentation temperature, is the effect of metabolic heat. Although the fermentations were performed in incubators set at 26 and 30 °C, respectively, the internal temperature of the broth was measured to be ~ 1 to 2 degrees higher; this could be due to the heat released by the metabolic activity of the yeast [27] and may have a negative impact on the yeast cells’ vitality and/or viability.

### Industrial strain construction and screening

The construction of a robust, temperature tolerant CBP yeast that can simultaneously express heterologous amylases and produce ethanol efficiently would yield more cost-effective ethanol production from starchy feedstocks. The demand for higher temperature fermentations began in the 1980s [28], with benefits including an easier ethanol extraction process more suited to fuel ethanol production, decreased operational costs (especially in regions with hot climates where cooling of fermentation vessels is required), improved hydrolysis conditions and reduced risk of contamination [29]. Currently, the fermentation temperature used in industry ranges between 30 and 34 °C [30] and it is desirable to select an appropriate industrial strain for CBP that is able to continue fermenting sugars above the recommended fermentation temperature.

**Fig. 2:**
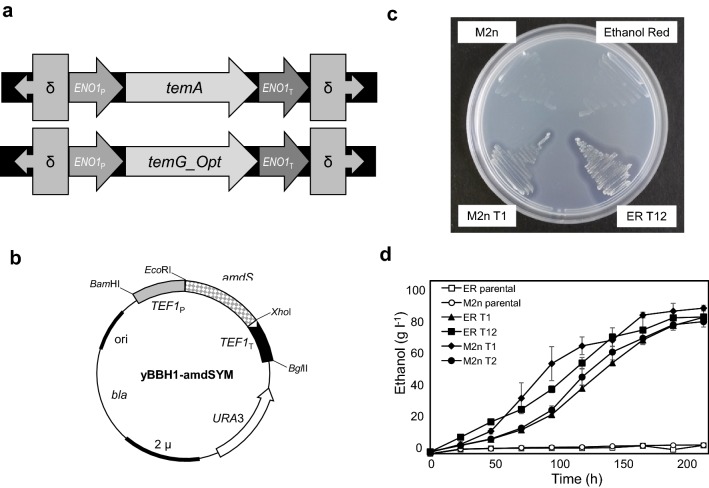
Construction of amylolytic *S. cerevisiae* M2n and ER industrial strains. The *ENO1 temA* and *temG_Opt* gene cassettes (**a**) were amplified using PCR and contained flanking regions homologous to the δ-integration sites. The *TEF1*_P_–*amdS*-*TEF1*_T_ cassette was cloned onto yBBH1 (**b**) to generate the yBBH1–amdSYM yeast expression vector. Soluble starch plate assays to visualise hydrolysis zones surrounding the recombinant strains (**c**), following incubation on soluble starch at 30 °C. The *S. cerevisiae* M2n and ER parental strains displayed no extracellular amylase activity. Ethanol concentrations produced by *S. cerevisiae* recombinant strains during fermentation in YPD with 5 g l^−1^ glucose and with 200 g l^−1^ corn starch at 30 °C (**d**) were compared to the parental strains. Data are the mean of three repeats showing standard deviation

The *S. cerevisiae* Ethanol Red™ strain (Fermentis, a division of S. I. Lesaffre, Lille, http://www.fermentis.com) was chosen as the main industrial expression host for this study (henceforth referred to as the ER strain), since it is predominantly applied in first-generation bioethanol production from corn and wheat [13, 31]. It is characterised by excellent fermentation capacity and yield, high robustness, stress- and thermo-tolerance [15]. For comparative purposes, the *S. cerevisiae* M2n South African distillery yeast strain was included as a parental strain [16]. The best performing amylase combination (*T. emersonii*’s *temA* and *temG_Opt* genes), identified through the fermentation screening process on raw corn starch (Fig. 1a), was selected for industrial strain transformation. The linear *ENO1*_P_-*temA*-*ENO1*_T_ and *ENO1*_P_–*temG_Opt*–*ENO1*_T_ DNA gene cassettes (Fig. 2a), flanked by the delta-(δ) sequences, were amplified by PCR using the primers listed in Table 3; δ-sequences are the long terminal repeats (LTRs) of the Ty1 and 2 retrotransposons and were targeted in the ER and M2n industrial strains’ genomes to generate multi-copy integrations [32, 33]. A markerless transformation method was also employed; the *amdS* selection marker gene (encoding for acetamidase) present on an episomal vector (Fig. 2b) was co-transformed with the gene cassettes (Fig. 2a), followed by plasmid curing (marker recycling).

**Table 3:** PCR primers designed and used in this study with the relevant restriction sites underlined (*Xho*I = ctcgag, *Bam*HI = ggatcc, *Bgl*II = agatct)

Primer name	Sequence (5′-3′)
ENOCASS-L	gtgcggtatttcacaccgcataggagatcgatcccaattaatgtgagttacctcactc
ENOCASS-R	cgggcctcttcgctattacgccagagcttagatct
amdSYMCas-L	ccgcgcgttggccgattcattaatccaggatccacatggaggcccagaataccctccttgac
amdSYMCas-R	gggcctcttcgctattacgccagagcttagatctcagtatagcgaccagcattcacatacttaa
Delta-ENO1_Promoter-L	tggaataaaaatccactatcgtctatcaactaatagttatattatcaatatattatcatatacggtgttaagatgatgacataagttatgagaagctgtcggatcccaattaatgtgagttacctcac
Delta-ENO1_Terminator-R	tgagatatatgtgggtaattagataattgttgggattccattgttgataaaggctataatattaggtatacagaatatactagaagttctcctcgaggatagatctcctatgcggtgtgaaataccgc

The industrial *S. cerevisiae* transformants were screened on SC-Ac plates containing 2% soluble starch (Fig. 2c) and only transformants that produced zones of hydrolysis were selected for further testing. PCR was used to confirm the integration of the respective *ENO1*_P_–*temA*-*ENO1*_T_ and *ENO1*_P_–*temG_Opt*-*ENO1*_T_ gene cassettes in transformants that produced prominent clearing zones (representing starch hydrolysis). From the 20 transformants selected for each strain, only *S. cerevisiae* strains ER T1, ER T12, M2n T1 and M2n T2 contained both integrated gene cassettes. These four strains were evaluated using liquid assays at both 30 and 37 °C to quantify the extracellular amylase activity on soluble and raw corn starch (Table 4). The higher the incubation temperature, the greater the extent of starch hydrolysis; at 37 °C, the *S. cerevisiae* ER T12 strain displayed the highest total amylase activity, 15.30 U ml^−1^ after 72 h, which was 3.8-fold higher than the *S. cerevisiae* M2n T1 strain’s activity of 3.99 U ml^−1^ (reducing sugar assay on soluble starch). The results from the raw starch assay performed at 37 °C also indicated that the *S. cerevisiae* ER T12 strain performed the best, displaying a total amylase activity of 1.38 U ml^−1^, which was significantly higher than the *S. cerevisiae* M2n T1 strain’s activity of 0.48 U ml^−1^, after 72 h. Mitotic stability was also tested and after 250 generations both ER T12 and M2n T1 strains were confirmed to be mitotically stable; these strains displayed hydrolytic ability on soluble corn starch.

**Table 4:** Soluble starch hydrolysing activities (U ml^−1^) of the industrial *S. cerevisiae* ER and M2n transformants expressing the *temG_Opt* glucoamylase and *temA* α-amylase originating from *T. emersonii* when grown in 2 × SC^−URA^ broth for 72 h

	2% raw starch		0.2% soluble starch	
	30 °C	37 °C	30 °C	37 °C
Total amylase activity (Reducing sugar assay^a^)				
ER T1	0.29 (0.10)	0.42 (0.09)	2.03 (0.35)	3.39 (0.20)
ER T12	0.99 (0.02)	1.38 (0.08)	9.11 (0.05)	15.30 (0.52)
M2n T1	0.33 (0.03)	0.48 (0.02)	2.21 (0.05)	3.99 (0.28)
M2n T2	0.20 (0.02)	0.29 (0.03)	1.28 (0.30)	2.46 (0.15)
Released glucose (Glucose kit assay^b^)				
ER T1	0.15 (0.03)	0.16 (0.04)	1.47 (0.23)	2.12 (0.18)
ER T12	0.44 (0.04)	0.48 (0.06)	4.43 (0.29)	6.32 (0.22)
M2n T1	0.27 (0.01)	0.29 (0.02)	1.11 (0.10)	1.59 (0.12)
M2n T2	0.16 (0.01)	0.17 (0.01)	0.65 (0.15)	0.96 (0.13)

The assays were performed at 30 °C and 37 °C in citrate buffer at pH 5 with raw and soluble corn starch. Parental strains did not give any starch-degrading activities. Values represent the mean of three repeats and standard deviations are reported in parentheses

^a^Reducing sugar assay detects all reducing sugars (monosaccharides and oligosaccharides)

^b^Glucose assay detects only glucose

These four industrial transformants were subsequently evaluated under fermentative conditions on raw corn starch (Fig. 2d). Significant differences in ethanol concentrations were noted during the first 96 h of fermentation at 30 °C (Fig. 2d). These differences followed similar trends to those observed during the liquid assays, with the *S. cerevisiae* ER T12 strain producing ethanol the quickest during the first 48 h. However, after 192 h, ethanol concentrations plateaued around ~ 80–90 g l^−1^. Both the assay and preliminary fermentation results showed that *S. cerevisiae* ER T12 and M2n T1 hydrolysed starch and fermented the sugars quicker than the *S. cerevisiae* ER T1 and M2n T2 strains (Table 4 and Fig. 2d) and were therefore selected for further evaluation.

### Next generation sequencing data analysis of *S. cerevisiae* ER T12 and M2n T1 genomes

Numerous studies have employed δ-elements for gene insertions because of their abundancy—several hundred δ-elements dispersed in the *S. cerevisiae* chromosomes [32]. These sites were chosen for the integration of the *temA* and *temG_Opt* genes because they created an opportunity to generate transformants with a varying number of gene copies, as well as different ratios of the amylolytic genes. Gene integration into targeted DNA sequences on the yeast’s chromosomes using the δ-sequences of the Ty retrotransposon allows for multiple gene integration and has assisted high expression levels in *S. cerevisiae* [16]. However, the δ-sites generate transformants with different expression efficiencies as the positions of the integrated cassettes are unknown and their numbers could vary substantially between transformants [34]. While Cho et al. [35] reported high copy numbers (maximum of 44 copies), most articles report less than 10 copies.

To identify the number of integrated amylase gene copies, the genomes of the *S. cerevisiae* ER T12 and M2n T1 amylase-producing strains were sequenced. The average number of paired-end reads (2 × 150 bp) for the strains was 3,750,382, resulting in a 106- and 169-fold genome coverage for *S. cerevisiae* M2n T1 and ER T12, respectively. The de novo assembly generated a draft genome of 11.7 and 11.6 Mb for *S. cerevisiae* M2n T1 and ER T12 strains, respectively. High-quality assemblies were composed by 251 and 159 scaffolds, with a N_*50*_ of 99334 and 188573 for *S. cerevisiae* M2n T1 and ER T12, respectively. Copy numbers for integrated genes in each genome were determined considering the ratio between the average coverage of selected housekeeping genes for *S. cerevisiae* and the average coverage of the integrated genes (Table 5).

**Table 5:** Average coverage of integrated *temA* and *temG_Opt* genes, as well as housekeeping genes into *S. cerevisiae* ER T12 and M2n T1 genomes

Genes	ER T12	M2n T1
*temA*	152 (*4.46*)	39 (*0.92*)
*temG_Opt*	245 (*7.20*)	41 (*0.99*)
*ALG9*	34	43
*TFC1*	34	42
*PGK1*	34	38
*ACT1*	35	44
Average housekeeping genes	34	42

Italic fonts report copy numbers integrated into each genome estimated considering the ratio between the average coverage of the integrated genes and the average coverage of the four housekeeping genes

*Saccharomyces cerevisiae* M2n T1 contains a single copy of each of the *temA* and *temG_Opt* genes, whilst the ER T12 strain has an estimated 4 *temA* and 7 *temG_Opt* copies. This finding is consistent with the higher enzymatic activities on soluble starch after 72 h at 37° by *S. cerevisiae* ER T12, which produced up to 3.8 and 3.9-fold the total amylase activity and released glucose, respectively, compared to *S. cerevisiae* M2n T1 (Table 4).

### Fermentations with the industrial *S. cerevisiae* strains and GSHE cocktail

**Fig. 3:**
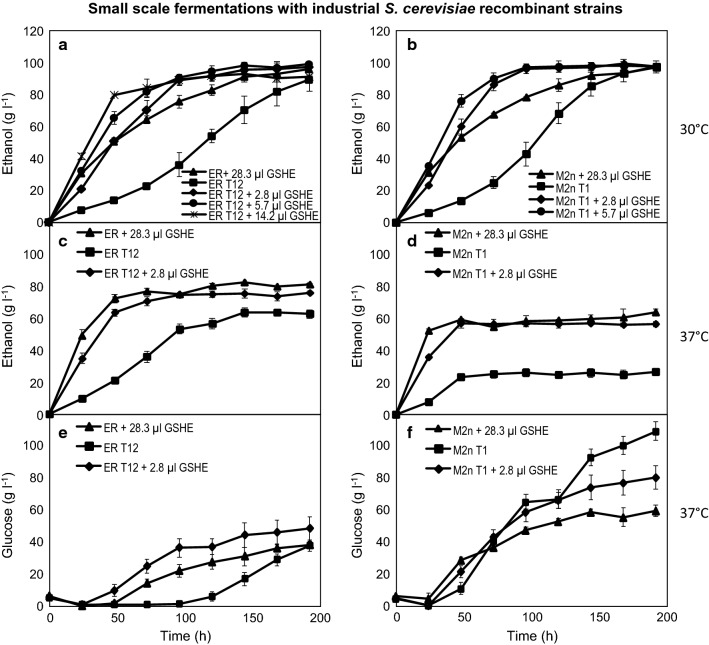
*S. cerevisiae* ER and M2n strains during fermentation in 100 ml fermentation bottles with YPD containing 5 g l^−1^ glucose and 200 g l^−1^ corn starch. Ethanol concentrations produced by ER (**a**) and M2n strains (**b**) at 30 °C, as well as ER (**c**) and M2n strains (**d**) at 37 °C. Glucose concentrations in fermentation broth with ER (**e**) and M2n strains (**f**) at an incubation temperature of 37 °C. Selected GSHE dosages (µl) were used to supplement the ER T12 and M2n T1 CBP fermentations, as well as provide SSF conditions for the ER and M2n parental strains. Data are the mean of three repeats showing standard deviation

CBP offers numerous advantages, however, at the start of a fermentation process the recombinant proteins still need to be produced by the yeast strain before substrate hydrolysis can accelerate. Therefore, enzyme supplementation was tested to enhance the rate of substrate hydrolysis to glucose during the first 24 h. The amylolytic *S. cerevisiae* ER T12 and M2n T1 strains were also compared to a simulated SSF control with the parental industrial strains so that a comparison to a raw starch CBP process could be made. The GSHE cocktail (used for enzyme supplementation) is a commercial raw-starch degrading preparation from DuPont Industrial Biosciences and the recommended GSHE dosage (100%) was calculated as 1.42 µl g^−1^ starch, according to the manufacturer’s specifications [36, 37]. The parental *S. cerevisiae* industrial strains supplemented with 28.3 µl GSHE per 100 ml fermentation (100% dosage of GSHE) represented the control experiment for each strain and a substrate loading of 200 g l^−1^ raw corn starch was used for all the fermentations (Fig. 3). At 30 °C, three different enzyme dosages in combination with the CBP strains were evaluated based on the percentage of the recommended enzyme loading: 2.8 µl (10%), 5.7 µl (20%) and 14.2 µl (50%—only for ER T12), while at 37 °C a 2.8 µl (10%) dosage of GSHE was evaluated with both *S. cerevisiae* ER T12 and M2n T1 strains.

At 30 °C, the ethanol profiles for the industrial *S. cerevisiae* parental strains were similar for the respective enzyme supplementation condition (Fig. 3a and b). By 48 h, the *S. cerevisiae* ER T12 strain supplemented with 2.8 µl GSHE (10% of the recommended dosage) had produced 50.78 g l^−1^ ethanol and also displayed an estimated carbon conversion of 50% (data not shown), compared to that of the control SSF process with the *S. cerevisiae* parental strain supplemented with 28.3 µl GSHE, which produced 50.57 g l^−1^ ethanol (Fig. 3a). Similarly, the *S. cerevisiae* M2n T1 strain supplemented with 2.8 µl GSHE produced 58.87 g l^−1^ ethanol, compared to the *S. cerevisiae* parental strain with 28.3 µl GSHE which produced 53.54 g l^−1^ ethanol, after 48 h (Fig. 3b). Therefore, for both strain backgrounds, the CBP supplemented conditions produced similar ethanol concentrations compared to the SFF control at 48 h, thereafter the CBP supplemented conditions displayed a higher rate of ethanol production.

After 96 h, the ethanol produced by the *S. cerevisiae* ER T12 strain supplemented with 2.8 µl GSHE (89.20 g l^−1^) was similar to the amount of ethanol produced by the *S. cerevisiae* ER T12 strain supplemented with 5.7 µl GSHE (90.82 g l^−1^) (Fig. 3a) and the estimated carbon conversion displayed was between 87 and 89% (data not shown). Under these two conditions, a significant increase in ethanol concentration was observed at 96 h, compared to the industrial *S. cerevisiae* control experiment (supplemented with a 100% GSHE loading), which produced 75.47 g l^−1^ ethanol and displayed an estimated carbon conversion of 74%. Therefore, the addition of 2.8 µl GSHE (10% of the recommended dosage) was sufficient to obtain results that were comparable to an SSF control. If the aim is to decrease the fermentation time, higher dosages of GSHE can be used in combination with the *S. cerevisiae* ER T12 strain. For instance, supplementing the CBP fermentation with 50% of the recommended dosage (14.2 µl GSHE) did not improve the final ethanol concentrations, but did result in a decreased fermentation time, with the maximum ethanol concentration being reached at 96 h, instead of 192 h. A higher enzyme loading thus contributed to increased ethanol productivity during the initial stage of fermentation at 30 °C, but the maximum ethanol concentrations achieved for the CBP supplemented experiments were similar ~ 95–97 g l^−1^ (Fig. 3a, b; Table 6).

**Table 6:** Product formation by *S. cerevisiae* ER and M2n strains after 192 h of fermentation at 30 °C and 37 °C in YPD media (containing 5 g l^−1^ glucose) and raw corn starch (200 g l^−1^), supplemented with selected GSHE dosages

	30 °C						37 °C					
*S.* *cerevisiae* strains	ER^a^	M2n^a^	ER T12	M2n T1	ER T12	M2n T1	ER^a^	M2n^a^	ER T12	M2n T1	ER T12	M2n T1
GSHE added (µl)	28.3	28.3	2.8	2.8	0	0	28.3	28.3	2.8	2.8	0	0
Substrate (g l^−1^)												
Raw starch	200	200	200	200	200	200	200	200	200	200	200	200
Glucose equivalent	208.5	208.5	208.5	208.5	208.5	208.5	208.5	208.5	208.5	208.5	208.5	208.5
Products (g l^−1^)												
Glucose	0.02	0.31	0.02	3.28	0.22	0.09	40.12	62.30	50.79	83.92	39.62	113.91
Glycerol	4.07	4.30	4.76	4.59	3.18	3.73	5.72	5.64	6.07	5.54	5.50	2.63
Acetic acid	0.00	0.00	0.90	0.31	0.64	0.00	0.83	0.66	0.95	0.39	1.28	0.95
Ethanol	95.90	97.34	97.16	97.84	89.35	98.13	81.30	63.99	75.99	56.82	62.64	26.96
Maltose	0.79	0.71	0.31	0.37	1.95	0.96	1.27	2.53	2.14	3.23	0.70	3.37
CO_2_	91.73	93.11	92.93	93.59	85.46	93.87	77.76	61.21	72.69	54.35	59.92	25.79
Total	192.51	195.77	196.07	199.98	180.79	196.78	207.00	196.33	208.63	204.25	169.66	173.62
Estimated carbon conversion (%)	92	94	94	96	87	94	99	94	100	98	81	83
Ethanol yield^b^ (% of theoretical yield)	92	93	93	94	86	94	78	61	73	55	60	26
Ethanol productivity^c^	0.50	0.51	0.51	0.51	0.47	0.51	0.42	0.33	0.40	0.30	0.33	0.14

^a^Parental strains under SSF conditions

^b^Ethanol yield (% of the theoretical yield) was calculated as the amount of ethanol produced per gram of available glucose (at a specific time point)

^c^Ethanol productivity was calculated based on ethanol concentrations produced per hour (g l^−1^ h^−1^)

During fermentation with the CBP industrial strains there was an initial “lag” phase in estimated carbon conversion up until 48 h. This was expected since the strains first had to adjust to the fermentation conditions and produce amylases de novo. However, the amylolytic CBP yeasts described in this study were able to continually replenish the recombinant enzymes in the fermentation broth and together with GSHE supplementation facilitated an overall increase in estimated carbon conversion (Table 6). On the other hand, GSHE are in abundance at the start of an industrial SSF cold hydrolysis set-up and rapidly produced glucose upon addition. However, the enzyme’s efficiency may decrease overtime due to their half-life. Results from this study clearly highlight the benefit of adding GSHE in combination with the amylolytic yeast strains, and limited enzyme supplementation provided the necessary boost to increase the rate of fermentation with the CBP yeast strains.

### Effect of fermentation temperature

At 30 °C, the final ethanol concentrations differed significantly between the *S. cerevisiae* M2n T1 and ER T12 strains under CBP conditions. The *S. cerevisiae* M2n T1 achieved a maximum ethanol concentration of 98.13 g l^−1^ after 192 h, which was significantly higher (*p *= 0.0054) (8.78 g l^−1^) than the *S. cerevisiae* ER T12 strain’s ethanol concentration of 89.35 g l^−1^ (Fig. 3a, b). However, at an incubation temperature of 37 °C, it was clear that the *S. cerevisiae* ER T12 strain had a greater fermentation vigour and was more tolerant to increasing ethanol concentrations, compared to the *S. cerevisiae* M2n T1 strain (Fig. 3c, d). Under CBP conditions (without enzyme supplementation), the *S. cerevisiae* ER T12 strain had a higher temperature tolerance and was able to ferment for longer at 37 °C (compared to the M2n T1 strain) and produced a > twofold increase in ethanol concentration after 192 h (Fig. 3c, d). Although the recombinant *S. cerevisiae* M2n T1 strain produced a higher ethanol yield at 30 °C, it was severely affected at an incubation temperature of 37 °C, where it reached an incomplete fermentation after 48 h (Fig. 3d). On average, glycerol concentrations were also higher at 37 °C (Table 6), signifying enhanced stress on both strains [14].

The extent of estimated carbon conversion displayed by the *S. cerevisiae* ER T12 strain (no GSHE supplementation) was similar (~ 81–87%) at the two fermentation temperatures (Table 6), while the estimated carbon conversion displayed by the *S. cerevisiae* M2n T1 strain was 11% higher at 30 °C, compared to the estimated carbon conversion at 37 °C (Table 6). Both the amylolytic *S. cerevisiae* ER T12 and M2n T1 strains had lower ethanol productivity at 37 °C, compared to at 30 °C and residual glucose levels were > 40 g l^−1^ at 37 °C (Fig. 3e, f, Table 6), which represented a large amount of unfermented glucose, especially for the *S. cerevisiae* M2n strains. Overall, results showed that thermotolerance played a major role in the fermentation vigour of industrial *S. cerevisiae* ER T12 and M2n T1 strains and affected the conversion of glucose to ethanol, thus supporting the observation in Fig. 1c with the Y294[TemG_Opt-TemA] strain.

### CBP industrial strains

There are currently no industrial amylolytic *S. cerevisiae* strains (co-expressing an α-amylase and glucoamylase gene) available for the conversion of starch to ethanol under CBP conditions [38] and few studies have successfully engineered *S. cerevisiae* ER for the expression of gene cassettes or adapted it for desired characteristics. Demeke et al. [15] developed a D-xylose fermenting strain, Wallace-Salinas and Gorwa-Grauslund [39] developed a strain capable of fermenting spruce hydrolysate and Stovicek et al. [40] introduced a xylose consumption pathway. To our knowledge, this study represents the first to engineer *S. cerevisiae* ER for the co-expression of both an α-amylase and glucoamylase gene for efficient raw corn starch conversion. It also represents the first study to investigate the effects of GSHE supplementation in combination with industrial amylolytic CBP yeast strains.

Compared to other studies, the industrial strains constructed in this study performed well on raw corn starch. Final ethanol concentrations were higher than those reported for the amylolytic haploid yeast strain, which produced 46.5 g l^−1^ of ethanol from 200 g l^−1^ of raw corn starch after 120 h of fermentation [41]. The novel amylolytic yeast strains presented here were superior in their ethanol production, producing 54.06 and 68.52 g l^−1^ ethanol for the *S. cerevisiae* ER T12 and M2n T1 strains, respectively, after 120 h (Fig. 3a, b), even with a much lower inoculum size (10% v v^−1^ was used in this study). Furthermore, since the recombinant amylases were secreted into the fermentation broth they can have increased physical contact with the starch granules, compared to other recombinant yeast that may display amylases on the cell’s surface [41]. Results also showed significant improvements when compared to the industrial *S. cerevisiae* M2n[TLG1–SFA1] and MEL2[TLG1–SFA1] amylolytic strains [16] that produced 64 g l^−1^ ethanol from 200 g l^−1^ raw corn starch (at a bioreactor scale), corresponding to 55% of the theoretical ethanol yield, as well as the *S. cerevisiae* Mnuα1[AmyA-GlaA] strain [19] that produced 65.83 g l^−1^ ethanol (after 10 days) representing 57% of the theoretical ethanol yield. Ethanol yields (% of the theoretical) obtained from the recombinant industrial strains in this study were > 85% (30 °C incubation temperature, Table 6) and thus represented a significant improvement on previously constructed CBP strains.

The cost of commercial enzyme addition has been estimated at 4.8 US cents per gallon, representing 8.3% of the total possessing costs in ethanol production from corn [42]. The amylolytic *S. cerevisiae* ER T12 and M2n T1 strains represent a novel alternative for lowering the enzyme dosage for raw starch hydrolysis, as well as being able to provide constant amylolytic activity for a continuous cold fermentation process. Furthermore, the use of amylolytic CBP yeast would allow for a simplified fermentation design, since pretreatment steps and costs can be bypassed [9].

## Conclusion

An improvement in the estimated carbon conversion of raw corn starch was achieved in this study and an incubation temperature of 30 °C enabled higher ethanol concentrations for the industrial strains, compared to fermentations at 37 °C. The amylolytic *S. cerevisiae* ER T12 and M2n T1 industrial strains, expressing the native α-amylase and codon optimised glucoamylase from *T. emersonii*, represent a suitable drop-in CBP yeast substitute for the existing cold fermentation process as they produced in excess of 80% of the theoretical ethanol yield. Although high-temperature fermentations are more practical for industrial ethanol production, results showed that ethanol- and thermo-tolerance are limiting factors with regards to constructing a CBP yeast for the industrial production of bioethanol. Therefore, future studies aimed at ethanol and temperature tolerance yeast are required to engineer a robust amylolytic CBP strain that can ferment at higher temperatures.

## Methods

### Media and cultivation conditions

All chemicals were of analytical grade and were obtained from Merck (Darmstadt, Germany), unless otherwise stated. *Escherichia coli* DH5α (Takara Bio Inc.) was used for vector propagation. The *E. coli* transformants were selected for on Luria–Bertani agar (Sigma-Aldrich, Steinheim, Germany), containing 100 μg ml^−1^ ampicillin and cultivated at 37 °C in Terrific Broth (12 g l^−1^ tryptone, 24 g l^−1^ yeast extract, 4 ml l^−1^ glycerol, 0.1 M potassium phosphate buffer) containing 100 µg ml^−1^ ampicillin for selective pressure [43].

The *S. cerevisiae* parental strains were maintained on YPD agar plates (g l^−1^:10 yeast extract, 20 peptone, 20 glucose and 15 agar) and *S. cerevisiae* Y294 transformants were selected for and maintained on SC^−URA^ agar plates (6.7 g l^−1^ yeast nitrogen base without amino acids (BD-Diagnostic Systems, Sparks, Maryland, USA), 20 g l^−1^ glucose and 1.5 g l^−1^ yeast synthetic drop-out medium supplements (Sigma-Aldrich, Steinheim, Germany) and 15 g l^−1^ agar). *S. cerevisiae* strains were aerobically cultivated on a rotary shaker (200 rpm) at 30 °C, in 125 ml Erlenmeyer flasks containing 20 ml double strength SC^−URA^ medium (2 × SC^−URA^ containing 13.4 g l^−1^ yeast nitrogen base without amino acids (BD-Diagnostic Systems), 20 g l^−1^ glucose and 3 g l^−1^ yeast synthetic drop-out medium supplements). Fermentation media for the *S. cerevisiae* Y294 strains comprised of 2 × SC^−URA^ containing 5 g l^−1^ glucose and 200 g l^−1^ raw corn starch (starch from corn—Sigma-Aldrich) [19, 24], whereas the medium for the industrial was YPD containing 5 g l^−1^ glucose and 200 g l^−1^ raw corn starch. Ampicillin (100 μg ml^−1^) and streptomycin (50 μg ml^−1^) were added to inhibit bacterial contamination. All cultures were inoculated to a concentration of 1 × 10^6^ cells ml^−1^, unless stated otherwise.

The industrial *S. cerevisiae* transformants were selected for on SC-Ac plates (SC plates with (NH_4_)_2_SO_4_ replaced by 0.6 g l^−1^ acetamide and 6.6 g l^−1^ K_2_SO_4_), containing 2% soluble corn starch. SC-Fac plates (SC media containing 2.3 g l^−1^ fluoroacetamide) was used to induce the plasmid curing of the yBBH1-amdSYM episomal vector from the transformants. The pH for SC-Ac and SC-Fac plates was adjusted to 6.0 with NAOH.

### Strains and plasmids

The genotypes of the bacterial and yeast strains, as well as the plasmids used in this study are summarised in Table 1.

### DNA manipulations

Standard protocols were followed for all DNA manipulations and *E. coli* transformations [43]. The enzymes used for restriction digests and ligations were purchased from Inqaba Biotec (Pretoria, South Africa) and used as recommended by the supplier. Digested DNA was eluted from 0.8% agarose gels using the Zymoclean™ Gel DNA Recovery Kit (Zymo Research, California, USA). The *ENO1*_P_–α-amylase-*ENO1*_T_ cassettes were amplified from the respective yBBH1–α-amylase plasmids (Table 1) using yeast mediated ligation (YML) cassette primers ENOCASS-L and ENOCASS-R (Table 3) and cloned into the *Bgl*II site of the yBBH1-glucoamylase plasmid (Fig. 1a). The *temA* and *temG_Opt* gene cassettes (containing the *ENO1* promoter and terminator) (Fig. 1c) were amplified through polymerase chain reaction (PCR) using the Delta-ENO1_Promoter-L and Delta-ENO1_Terminator-R primers (Table 3), together with the yBBH1-TemA and yBBH1-TemG_Opt plasmids [20], respectively, as templates.

The *TEF1*_P_-*amdS*-*TEF1*_T_ gene cassette was amplified from pUG-amdSYM through PCR using the amdSYMCas primers (Table 3) and cloned onto yBBH (digested with *BamH*I and *Bgl*II to remove the *ENO1*_P_ and *ENO1*_T_) to yield plasmid yBBH1-amdSYM (Fig. 2b). The *Ashbya gossypii TEF1* promoter regulated the expression of the acetamidase-encoding gene (*amdS*) for the selection of transformants on SC-Ac plates. The yBBH1-amdSYM plasmid was retrieved from the *S. cerevisiae* Y294[amdSYM] strain and transformed into *E. coli* DH5α to obtain a high concentration of plasmid DNA. Plasmid DNA was isolated using the High Pure Plasmid Isolation kit (Roche, Mannheim, Germany). DNA sequence verification was performed by the dideoxy chain termination method, with an ABI PRISM™ 3100 Genetic Analyser (CAF, Stellenbosch University, South Africa).

### Yeast transformations

The *S. cerevisiae* Y294 strain was grown overnight in 5 ml YPD broth and prepared according to [35] and transformed by means of electroporation using a Bio–Rad system (GenePluserXcell TM, Bio-Rad, Hercules, California, USA). After electroporation, 1 ml of YPDS (YPD supplemented with 1 M sorbitol) was immediately added to the cuvette. Cultures were incubated at 30 °C for 1 h prior to plating out onto SC^–URA^ plates containing 2% soluble corn starch. Plates were incubated at 30 °C for 2–3 days and then transferred to 4 °C for 24 h to allow the starch to precipitate.

Electro-competent industrial yeast cells were prepared in the same manner. For the transformation of industrial strains, amylase DNA (linear *temA* and *temG_Opt ENO1* cassettes) were simultaneously transformed into the yeasts genomes using the yBBH1-amdSYM episomal vector, which contained the *amdS* selection marker (Fig. 2b). After electroporation, 1 ml of YPDS was immediately added to the cuvettes and the cells were incubated at 30 °C for 3 h. Transformants were selected for by plating the transformation mix on to SC–Ac plates containing 2% starch [adapted from 17] and incubated at 30 °C for 24 h. The integration of the linear DNA expression cassettes into the yeast genome was confirmed by PCR using gene-specific primers [20] and gene copy numbers were estimated using whole-genome sequencing.

### Genomic DNA extraction and library sequencing

Genomic DNA was extracted from overnight yeast cultures according to PowerSoil^®^ DNA Isolation Kit (MO BIO laboratories Inc., Carlsbad, CA USA). An additional cleaning step with Phenol: Chloroform: Isoamyl Alcohol (25: 24: 1) (Sigma-Aldrich) was performed before DNA isolation. Genomic libraries were generated using the TruSeq DNA PCR-Free Library Prep Kit (Illumina Inc., San Diego CA) and Covaris S2 (Woburn, MA) for a 550-bp average fragment size. Libraries were loaded onto the flow cell provided in the NextSeq500 Reagent kit v2 (150 cycles) (Illumina Inc., San Diego CA) and sequenced on a NextSeq500 (Illumina Inc., San Diego CA) platform with a paired-end protocol and read lengths of 151 bp at the CRIBI Biotechnology Center (Padova, Italy) to determine the copy number of the integrated *temA* and *temG_Opt* genes.

### Next-generation sequencing data analysis

Raw reads were filtered using Trimmomatic version 0.33 (leading:35 trailing:35 sliding window:4:15 headcrop:35 minlen:100). The de novo assembly was performed using SPAdes version 3.9 (with option -k 21,33,55,77) [44]. High quality-filtered reads were aligned to assembled genomes using bowtie2 [45]. The assembled genomes were used to create a local database for BLAST analysis. All sequences of the integrated genes *temA* and *temG_Opt* and housekeeping genes (*ACT1*, *ALG9*, *PGK1*, *TFC1*) were used as queries for BLAST search against *S. cerevisiae* M2n T1 and ER T12 strains, independently. Copy numbers for integrated genes in each genome were determined by taking the ratio of average coverage of the integrated genes to average coverage of all scaffolds [46]. The coverage (the depth of sequencing) was calculated using BBMap in BBTools (http://sourceforge.net/projects/bbmap). Moreover, the estimation of the integrated copy numbers was assessed considering the ratio between the average coverage of selected housekeeping genes for *S. cerevisiae* and the average coverage of the integrated genes. Statistically similar copy numbers were determined considering the ratio of integrated genes’ average coverage to the average coverage of both all scaffolds and selected housekeeping genes. The genome assembly of *S. cerevisiae* M2n T1 and ER T12 was deposited at GenBank under the accession number SKCB00000000 and SKCC00000000, respectively. The versions described in this paper are version SKCB01000000 and SKCC01000000, respectively.

### Activity assays

Industrial yeast transformants were cultured in 20 ml 2 × SC^−URA^ media (inoculated at a concentration of 1 × 10^7^ cells ml^−1^), in 125 ml Erlenmeyer flasks with agitation at 200 rpm and sampling at 24-h intervals. The assays for quantitative analysis of amylase activity were performed as described by [20]. The supernatant was used to colourimetrically assess (xMark™ Microplate Spectrophotometre, Bio-Rad, San Francisco, USA) the total extracellular amylase activity levels using the reducing sugar assay with glucose as standard [47]. The glucoamylase activities (released glucose) were determined according to the method described by Viktor et al. [19]. Enzymatic assays were performed in triplicate at pH 5 and at 30 and 37 °C, using 0.05 M citrate buffer. Enzymatic activity was expressed as U ml^−1^ supernatant, with one unit defined as the amount of enzyme required to release one µmole of glucose per minute, under the described assay conditions. Soluble starch assays were performed using 0.2% soluble (autoclaved) corn starch, while raw starch assays were performed using 2% raw corn starch [48]. To determine glucose equivalents released from raw starch by engineered laboratory strains, the glucose and maltose concentrations were determined using HPLC, as described below under “Analytical methods and calculations”.

### Evaluation of mitotic stability of the industrial transformants

To study mitotic stability of the obtained ER T12 and M2n T1 strains, the transformants were grown in sequential batch cultures using a method adapted from [48]. The strains were cultivated in non-selective YPD broth (5 mL) on a rotating wheel and transferred (1% v v^−1^) to fresh YPD after glucose depletion. After 250 generations, recombinant strains were plated onto YPD and incubated at 30 °C for 24 h. Up to 100 colonies for each transformant were replicated onto SC^–URA^ plates containing 2% soluble corn starch. The stable transformants displayed hydrolytic activity on the starch plates after 24 h.

### Marker recycling

Plasmid curing was performed on the industrial recombinant strains according to [17]. The removal of the yBBH1-amdSYM plasmid containing the acetamide marker was achieved by growing cells overnight in 5 ml liquid YPD and transferring 20 µl to a 125 ml Erlenmeyer flask containing 10 ml SC-Fac media. Marker-free single colonies were obtained by plating 100 µl of culture on SC-Fac solid media containing 2% soluble corn starch.

### Fermentations

*Saccharomyces cerevisiae* Y294 precultures were cultured in 60 ml 2 × SC^−URA^ medium in 250 ml Erlenmeyer flasks, whereas industrial *S. cerevisiae* ER and M2n precultures were cultivated similarly in YPD medium, for small scale fermentations. Flasks were incubated at 30 °C with agitation at 200 rpm. Fermentations with the *S. cerevisiae* Y294 strains were performed at an incubation temperature of 30 °C according to [24], while fermentations with the industrial *S. cerevisiae* yeasts were performed at both 30 °C and 37 °C in YPD medium (containing 5 g l^−1^ glucose) with a 10% inoculum. The substrate loading for all fermentations was 200 g l^−1^ corn starch (183.3 g l^−1^ dry weight). The exogenous GSHE cocktail used to supplement the fermentation process was STARGEN 002™ (referred to as GSHE in this study) obtained from Dupont Industrial Biosciences (Palo Alto, California, USA) with an activity minimum of 570 GAU gm^−1^ (http://www.genencor.com) and used according to the manufacturers instructions. STARGEN 002™ contains *Aspergillus kawachii* α–amylase expressed in *Trichoderma reesei* and a glucoamylase from *T. reesei* that work synergistically to hydrolyse granular starch to glucose [37].

#### Bioreactor fermentations

*Saccharomyces cerevisiae* Y294 precultures were cultivated in 120 ml 2 × SC^−URA^ media in 500 ml Erlenmeyer flasks at 30 °C with agitation at 200 rpm. Bioreactor fermentations were performed in a 2-l MultiGen Bioreactor (New Brunswick Scientific Corporation, New Jersey, USA) containing 2 × SC^−URA^ media supplemented with 200 g l^−1^ raw corn starch and 5 g l^−1^ glucose as carbohydrate source. A 10% (v v^−1^) inoculum was used in a total working volume of 1-l. Fermentations were carried out at incubation temperatures of 26 °C and 30 °C, with stirring at 300 rpm and daily sampling through a designated sampling port. All fermentation experiments were performed in triplicate.

#### Analytical methods and calculations

Ethanol, glucose, maltose, glycerol and acetic acid concentrations were quantified using High-performance liquid chromatography (HPLC) according to [24]. The theoretical CO_2_ yields were calculated according to [16]. The glucose equivalent is defined as the mass of glucose resulting from the complete hydrolysis of starch, i.e. 1.11 grams of glucose per gram of starch. The available carbon (mol carbon in 100% hydrolysed substrate) was calculated based on the available glucose (glucose equivalent used was 208.5 g l^−1^, therefore, total mol carbon equals 6.95). The estimated carbon conversion is defined as the percentage starch converted on a mol carbon basis (Eq. 1). The estimated carbon conversion (as a percentage) was calculated from ethanol, glucose, maltose, glycerol, acetic acid and CO_2_ concentrations using the following equation:

Equation 1: Estimated carbon conversion (%)

1 $$\frac{{\left[ {\left( {{\text{maltose}} \times \frac{12}{342}} \right) + \left( {{\text{glucose}} \times \frac{6}{180}} \right) + \left( {{\text{glycerol}} \times \frac{3}{92}} \right) + \left( {{\text{acetic acid}} \times \frac{2}{60}} \right) + \left( {{\text{carbon dioxide}} \times \frac{1}{44}} \right)} \right] }}{\text{mol carbon}} \times 100$$

The ethanol yield (% of the theoretical yield) was calculated as the amount of ethanol produced per gram of available glucose. The ethanol productivity was calculated based on ethanol concentration produced per h (g l^−1^ h^−1^).

### Statistical analysis

Measurements represent the mean of three repeats. Data was analysed using the Student’s *t* test to determine significant differences between recombinant yeast strains.

## Data Availability

The datasets generated during the current study are available in the GenBank repository, accession numbers provided in text.

## Abbreviations

bp: base pair
CBP: consolidated bioprocessing
cDNA: copy-deoxyribonucleic acid
DDGS: distiller’s dried grains with solubles
DNA: deoxyribonucleic acid
GSHE: granular starch hydrolysing enzyme
HPLC: high performance liquid chromatography
PCR: polymerase chain reaction
SDS-PAGE: sodium dodecyl sulphate polyacrylamide gel electrophoresis
SC: synthetic complete
SC^−URA^: synthetic complete lacking uracil
SSF: simultaneous saccharification and fermentation
YPD: yeast extract, peptone, dextrose
YML: yeast mediated ligation
